# The Effect of *Spirulina platensis versus* Soybean on Insulin Resistance in HIV-Infected Patients: A Randomized Pilot Study

**DOI:** 10.3390/nu3070712

**Published:** 2011-07-18

**Authors:** Azabji-Kenfack Marcel, Loni G. Ekali, Sobngwi Eugene, Onana E. Arnold, Edie D. Sandrine, Denis von der Weid, Emmanuel Gbaguidi, Jeanne Ngogang, Jean C. Mbanya

**Affiliations:** 1 Department of Physiological Sciences and Biochemistry, Faculty of Medicine and Biomedical Sciences, University of Yaounde 1, Melen Street, Cameroon; Email: ediesandrine@yahoo.fr (E.D.S.); jngogang@yahoo.fr (J.N.); 2 Department of Internal Medicine, Faculty of Medicine and Biomedical Sciences, University of Yaounde 1, Melen Street, Cameroon; Email: eloni2000@yahoo.com (L.G.E.); sobngwieugene@yahoo.fr (S.E.); onana_arnold@yahoo.fr (O.E.A.); jcmbanya@hopitcam.net (J.C.M.); 3 Antenna Technologies Foundation, Rue de Neuchâtel 29 1201 Geneva, Switzerland; Email: dweid@antenna.ch; 4 PPSAC/KfW/OCEAC, Yaounde, Cameroon; Email: eac_gbaguidi@yahoo.fr

**Keywords:** spirulina, soybean, insulin resistance, HIV, HAART

## Abstract

HIV-infected patients develop abnormalities of glucose metabolism due to the virus and antiretroviral drugs. Spirulina and soybean are nutritional supplements that are cheap, accessible in our community and affect glucose metabolism. We carried out a randomized study to assess the effect of *Spirulina platensis versus* soybean as a food supplement on HIV/HAART-associated insulin resistance (IR) in 33 insulin-resistant HIV-infected patients. The study lasted for two months at the National Obesity Centre of Cameroon. Insulin resistance was measured using the short insulin tolerance test. Physical activity and diet did not change over the study duration. On-treatment analysis was used to analyze data. The Mann-Whitney U test, the Students T test and the Chi square test were used as appropriate. Curve gradients were analyzed using ANCOVA. Seventeen subjects were randomized to spirulina and 16 to soybean. Each received 19 g of supplement daily. The follow up rate was 65% *vs.* 100% for spirulina and soybean groups, respectively, and both groups were comparable at baseline. After eight weeks, insulin sensitivity (IS) increased by 224.7% *vs.* 60% in the spirulina and soybean groups respectively (*p* < 0.001). One hundred per cent *vs.* 69% of subjects on spirulina *versus* soybean, respectively, improved their IS (*p* = 0.049) with a 1.45 (1.05–2.02) chance of improving insulin sensitivity on spirulina. This pilot study suggests that insulin sensitivity in HIV patients improves more when spirulina rather than soybean is used as a nutritional supplement. Trial registration: ClinicalTrials.gov identifier NCT01141777.

## 1. Introduction

Even though antiretroviral therapy (ART) has dramatically improved the health of people living with the human immunodeficiency virus (HIV) [1], the prospect of maintaining patients long term on highly active antiretroviral therapy (HAART) can be severely restricted by the development of serious long term metabolic complications. These abnormalities include insulin resistance (IR), dyslipidemia and changes in body fat distribution [2,3,4,5,6,7,8]. Insulin resistance leads to dyslipidemia [9] and precedes significant changes in body weight and fat redistribution in HAART-treated patients [10]. 

Nutritional intervention [11,12,13] has been used successfully as a means of treating subjects with insulin resistance and diabetes either alone or in combination with physical activity [14] and/or drugs. 

Over the years, many dietary supplements have appeared in the market and have gained widespread use. The WHO projects that spirulina will become one of the most curative and prophylactic components of nutrition in the 21st century [15,16]. Preliminary studies suggest that it improves glycemic control, lowers cholesterol levels and reduces blood pressure [17,18] in diabetics. It is also cheap and easily available in our community and some researchers have even termed it a “superfood” [19]. 

On the other hand, soybean has been used for several centuries by the Asians and is well known as a dietary supplement with beneficial effects on human health. It is also cheap and easily available in our community. Studies have shown differing effects on insulin sensitivity and glucose metabolism in animals and humans [20,21,22]. Data on the health effects of spirulina and especially soybeans in African populations are rare despite its wide consumption within this population.

Given the fact that access to ART is increasing in Sub Saharan Africa, the prevalence of HAART-associated metabolic disorders is expected to rise in the coming years. There is therefore a need to research nutritional supplements that are cheap, easily available and can be used in the treatment and/or prevention of these metabolic abnormalities given the low purchasing power in this region. 

We therefore carried out this pilot study to evaluate the effect of these two supplements on HIV/HAART-associated insulin resistance (IR).

## 2. Experimental Section

The procedure used was in accordance with the guidelines of the Helsinki Declaration on human experimentation [23]. The study was approved by the National Ethics Committee of Cameroon (Authorization number 036/CNE/DNM/07). The purpose of the study was carefully explained and a written consent obtained from each subject before inclusion.

### 2.1. Participants

From March 2008 to January 2009, a total of 143 HIV-infected subjects were recruited at the Day Care Hospital of the Central Hospital of Yaounde. Subjects had been previously diagnosed as infected with HIV and followed up at the day care hospital. 

Adults confirmed as infected with HIV who agreed to participate in the study were included. All those with an acute intercurrent infection, on drugs that can modify the glucose or lipid profile other than HAART (steroids, insulin, *etc.*), actively taking tobacco, with renal failure (calculated creatinine clearance <60 mL/min), pregnant or known to be living with diabetes were excluded. 

### 2.2. Protocol

#### 2.2.1. Randomization, Treatment Allocation and Follow-Up

Subjects were allocated to treatment groups by simple randomization. Pieces of paper were marked “spirulina” or “soya beans” and then sealed. These papers were put in a box in equal numbers. Each subject blindly selected a sealed piece of paper from the ballot box at random and then handed it to the person in charge of treatment allocation who then recorded the treatment group in a register that he kept confidential and then administered the appropriate treatment to the subject. At every point in time, the sealed papers were always in equal numbers in the box so as to give each subject a 50% chance of belonging to either group. When the first group was completed, all the remaining subjects were allocated to the second group.

Spirulina was supplied by Antenna Technologies (Geneva, Switzerland) and soya beans were obtained from TANTY Ltd. (Yaounde, Cameroon). This spirulina was grown in the Republic of Equator and then packaged in Switzerland.

**Table 1:** Composition of spirulina and soya beans used in the trial.

	Spirulina	Soybean
	***Quantity per* 100 g**	***Quantity per* 100 g**
**Protein**	65 g	27 g
**Carbohydrates**	15 g	58 g
**Lipids**	6 g	8.6 g
**Calcium**	1000 mg	106.3 mg
**Iron**	180 mg	6.3 mg
**Magnesium**	400 mg	-
**Minerals**	7 mg	-

Antenna Technologies is specialized in the growth, transformation and distribution of high quality spirulina while TANTY Ltd. is a Cameroonian company specialized in the growth, transformation and distribution of soya bean products. The composition of each supplement is shown above in Table 1. The supplements were given as powder (spirulina green, soya beans white-brown) and subjects took 19 g daily with meals and were also asked to continue their normal daily activities and diet without modification. To monitor these parameters, a questionnaire on diet and physical activity was established and administered to each subject before and at the end of the trial. 

Follow-up visits took place every two weeks after the onset of the trial. During these visits the supplements were replenished and compliance was evaluated. To do this, subjects were given a form which they had to fill every day after having taken the supplements. The number of intakes skipped every 2 weeks as recorded in these forms was noted down.

#### 2.2.2. Clinical Assessment

Eligible subjects were randomized into two groups; spirulina and soya bean groups, and were each seen every two weeks for a total duration of 2 months. The duration of two months was chosen based on previous studies that showed significant effects of spirulina on glucose homeostasis within this period of time [17,18]. Subjects were asked to continue their diet and physical activity as usual. 

At study entry, patients had been fasting for at least eight hours overnight. The same operator noted the age and ART treatment regiment and duration of each eligible participant and evaluated their daily physical activity and diet using a quantitative questionnaire and assessed anthropometric data. 

Anthropometric measurements included weight, height, body mass index (BMI), waist circumference (WC) and total adiposity. Body height and weight (electronic weighing device, LAICA^®^, Italy) were measured without shoes and in light clothing, respectively. BMI was measured as the ratio between the weight and the square of the height (kg/m^2^). WC was measured as the circumference of the torso midway between the imaginary line that joins the twelfth rib to the iliac crest. Total adiposity was indirectly measured using a body fat monitor (Quantum III, RJL Systems, USA). Of the four electrodes emanating from the monitor, two were placed on the right hand and two on the right foot with the patient lying supine. Total body fat and lean body mass were calculated after introducing the resistance measured by the monitor into the NHANES III formulae [24]. All these measurements were repeated after 2 months of intervention.

#### 2.2.3. Biochemical Assays

Insulin sensitivity was measured by the short insulin tolerance test (SITT) using the slope of blood glucose concentration from 3 to 15 min after a low dose intravenous bolus of rapid insulin. In particular, 0.1 IU/kg of rapid insulin was injected intravenously after the fasting blood glucose had been measured. Glycemia was then recorded every 3 min for a total duration of 15 min using the HEMOCUE 201+ glucose analyzer (Angelholm, Sweden). The faster the decline in glucose concentration, the more insulin-sensitive the subject is. The slope of the linear decline in plasma glucose, which is the insulin sensitivity index (KITT) was calculated by dividing ln 2 (0.693) by the plasma glucose half-life (50% from baseline) [25].

          KITT = (0.693/*t*_1/2_) × 100

where *t*_1/2_ represents the half-life of the decrease in plasma glucose. Given that this test is population dependent and that insulin sensitivity has a normal distribution, we defined as insulin-resistant, all subjects with insulin sensitivity index (KITT) within the first tertile of the insulin sensitivity distribution of the study population. Subjects with KITT in the last tertile of the insulin sensitivity distribution were considered as insulin-sensitive while all those with KITT in the middle tertile were considered as having intermediate insulin sensitivity. 

Five (5) mL of whole blood was collected from a peripheral vein for lipid assay. The blood was centrifuged at 4000 rev/min at 4 °C and the serum obtained was stored at −20 °C until analysis. Total cholesterol (TC) and triglycerides (TG) were measured by the LISA 380 Plus automat (Hycel Diagnostics, France). All these measurements were repeated eight weeks after onset of intervention. 

#### 2.2.4. Outcome Measures

Given that the main objective of this study was to determine the effect that spirulina has on insulin resistance associated with ART among HIV-infected people, the primary outcome was the percentage difference in change in insulin sensitivity between the two groups at the end of eight weeks of intervention. The secondary outcome was the percentage of subjects who improved insulin sensitivity by the end of the study, compared between the two groups.

#### 2.2.5. Sample Size

The sample size for the study was calculated from an expected difference in change in insulin sensitivity of 20% between the two groups at the end of the study, using the mean insulin sensitivity, M, of a healthy Cameroonian population (M = 14.3 ± 2.2 mg/kg/min) [26]. Choosing α (two-sided) at 0.05 and a statistical power of 80%, the minimum sample size was 10 IR subjects per group. The calculations are given below [27]. 

The standardized difference, 

          *d* = target difference/standard deviation

          *d* = 2.86/2.2 = 1.3

For two equal groups, the sample size per group is given by the formula below [27]: 

          *n* = (2/*d*^2^) × *C_p,power_*

where *C_p,power_* is a constant defined by the value chosen for the power and *p* value, available in statistical tables. With a power of 80% and a *p* value of 0.05, the constant is 7.9 [27].

Thus the sample size per group, 

          *n* = (2/1.3^2^) × 7.9 

          *n* = 9.35 subjects

This number was rounded up to a minimum of 10 insulin-resistant subjects per group.

### 2.3. Statistical Methods

Collected data was recorded on a preformed questionnaire which was computed and validated using the Epi Info software, Version 3.4.3. To ensure accuracy of data, all computerized entries were further checked against those on paper, item by item. Finally, data were exported to Microsoft Excel 2003, the Statistical Package for Social Sciences software, SPSS (Version 13.0) and Graphpad Prism (Version 5) for further analysis.

Results are expressed as frequencies or mean and standard deviation. The Mann-Whitney U test and the Student’s *T* test were used to compare continuous data as appropriate. For categorical variables, the Chi square test was used. Gradients of curves were compared using analysis of covariance (ANCOVA). All *p*-values less than 0.05 were considered statistically significant for all analyses. 

## 3. Results and Discussion

**Figure 1:**
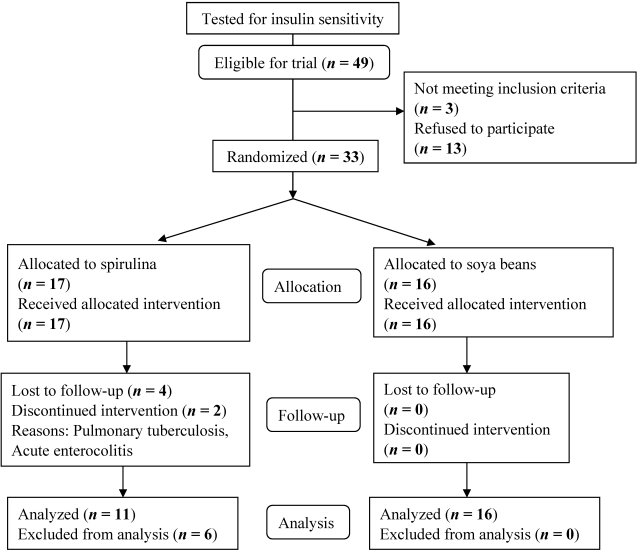
Participant flow in the course of the study.

Of the 143 HIV-infected subjects assessed for insulin sensitivity, 49 were insulin resistant (KITT ≤ 1.620%/min), 47 had intermediate insulin sensitivity (1.620 < KITT ≤ 1.683) and 47 were insulin sensitive (KITT > 1.683). Of the 49 subjects eligible for the trial, 3 did not meet inclusion criteria while 13 refused to participate. Seventeen were therefore randomized to spirulina and 16 to the soybean group. Two subjects were discontinued treatment in the spirulina group due to acute intercurrent infection, 2 withdrew their consent while 2 loses to follow up were registered in the spirulina group giving an overall follow up rate of 82% (Figure 1).

Physical activity and diet did not change over the study duration. The mean number of days during which the supplement was taken was significantly lower for spirulina than for soya beans (44.8 ± 19.1 *versus* 59.4 ± 1.5 days, *p* = 0.005). This could be explained by the fact that all subjects on spirulina as opposed to none on soya bean complained about the taste, saying that it was not palatable. This difference in taste could account for the significantly lower compliance demonstrated in the spirulina group and partly for the number of losses to follow up observed (65% follow up rate in spirulina *versus* 100% follow up rate in soya beans). But despite the loss to follow up and lower number of days of intake registered in the spirulina group (45 days), the effect on insulin sensitivity was significant. This mean duration of 45 days is similar to the duration of spirulina intake in a Mexican study done to assess the impact of spirulina on blood pressure [18]. However, we recommend that future studies should use spirulina forms which are more palatable to improve compliance.

After randomisation, the spirulina and soya bean groups were comparable for all anthropometric and biological parameters that were measured (Table 2). 

**Table 2:** Baseline characteristics of subjects.

Characteristics		Spirulina (*n* = 17)	Soya bean (*n* = 16)	*p* value
***Demographic and Clinical***				
Sex, *n*	Men	4	3	0.74
	Women	13	13	
Treatment regiment	ZDV+	12	12	0.95
	D4T+	4	3	
	Treatment naive	1	1	
Age, mean (SD), years		36 (11)	39 (7)	0.45
HAART duration, mean (SD), months		22 (14)	27 (17)	0.23
BMI, mean (SD), kg/m^2^		23.8 (3.2)	24.7 (2.8)	0.40
Systolic BP, mean (SD), mmHg		127 (17)	121 (11)	0.20
Diastolic BP, mean (SD), mmHg		78 (10)	80 (7)	0.63
Waist Circumference, mean (SD), cm		82.2 (9.1)	82.3 (7.3)	0.98
Fat-free mass, mean (SD), kg		38.2 (11.5)	37.1 (10.9)	0.78
Percent Body Fat, mean (SD), %		39.1 (17.4)	41.6 (16.3)	0.45
***Biological***				
CD4 count, median (Q1–Q3), µL		277 (75.5–472.3)	226 (112–402)	1.0
Total cholesterol, mean (SD), g/L		1.7 (0.49)	1.8 (0.43)	0.54
Triglycerides, mean (SD), g/L		0.7 (0.53)	0.6 (0.28)	0.77

Abbreviations: *n*, number of subjects; SD, Standard deviation; HAART, Highly active antiretroviral therapy; BMI, Body mass index; BP, Blood pressure; ZDV+, Zidovudine present; D4T+, Stavudine present; Q1–Q3, 25th–75th percentile.

**Figure 2:**
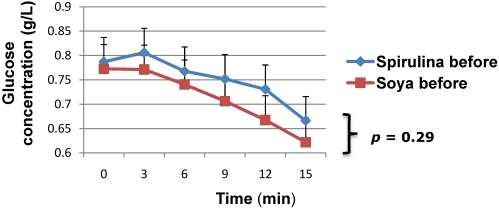
Insulin sensitivity at baseline compared between the two groups.

The rate of blood glucose decline during the SITT, which reflects insulin sensitivity (Figure 2), was also comparable between the two groups (−0.81%/min *vs.* −1.05%/min, *p* = 0.29) at baseline.

**Figure 3:**
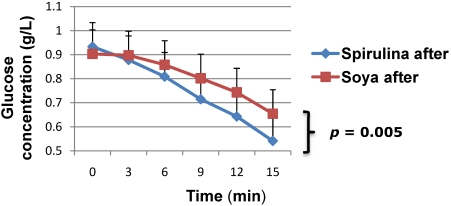
Insulin sensitivity compared between the two groups after eight weeks.

After 2 months of intervention, the rate of blood glucose decrease during SITT was significantly greater in the spirulina group (−2.63%/min *vs.* −1.68%/min, *p* = 0.005) compared to the soyabeans group (Figure 3). 

**Figure 4:**
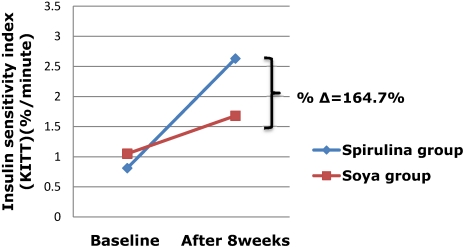
Variation of insulin sensitivity during the trial.

The sensitivity of insulin increased by 224.7% in those taking spirulina compared with 60% in those on soya (*p* < 0.001), giving a 164.7% difference in change of insulin sensitivity between the two groups at the end of the study (Figure 4).

**Figure 5:**
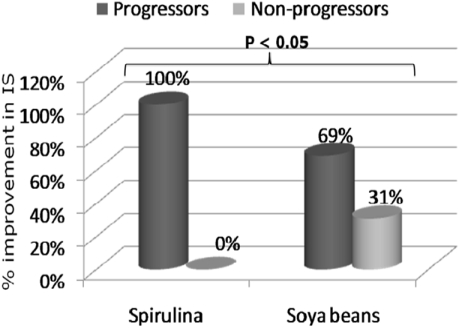
Improvement of insulin sensitivity by treatment group at the end of the trial.

In Figure 5, we compared the number of subjects who changed insulin sensitivity category in each group at the end of the intervention. All those who moved from a lower to a more insulin sensitive category were called progressors while all those who did not change category or moved to a lower category were called non progressors. After 2 months of spirulina supplementation as shown in Figure 5, 100% (11) of IR HIV-infected subjects improved their sensitivity to insulin while 31% (5) of subjects on soya beans had no improvement in their insulin sensitivity (*p* = 0.049). 

For subjects taking spirulina, there was a 1.45 increase in the chance of improving insulin sensitivity compared to soya beans (1.05 < RR < 2.02).

To our knowledge, this is the first study in which the effect of spirulina intake on insulin sensitivity has been measured. The significant increase in insulin sensitivity following spirulina intake could be mediated by the immune modulatory effect of spirulina. Park *et al.* had previously demonstrated that when human subjects were placed on 8 g of spirulina per day for 4 months, there was a marked reduction in the circulating levels of interleukin 6 [28]. In our study, subjects on spirulina had a reduction of their waist circumference as opposed to those on soya bean (see Table 3). A high waist circumference is associated with increased visceral abdominal fat. In HIV lipodystrophy, visceral abdominal fat is significant as it produces more IL-6 than subcutaneous fat. IL-6 contributes to IR by inhibiting insulin-signaling molecules like insulin receptor substrate 1 [29]. When this happens, the cascade of intracellular downstream reactions that are responsible for causing translocation of GLUT 4 to the cell surface, vital for glucose uptake in muscles and adipocytes, is inhibited. With a decrease in waist circumference, there could be a lower production of IL-6 in subjects on spirulina. This could partially explain the marked improvement in insulin sensitivity registered in the spirulina group despite the non-significant change in waist circumference. Interestingly, Park *et al.* observed that the change in the waist to hip ratio, used as a surrogate for abdominal obesity in their study, was not significant at the end of their study [28]. This is similar to our results where the WC, which is a surrogate for abdominal obesity did not show a significant decrease at the end of the study. This could indicate that minor changes in abdominal obesity can have significant changes biologically with respect to IL-6 dynamics. In our subjects, the fat-free mass of subjects on spirulina also increased while that of subjects on soybean decreased (Table 3). The muscle is a major component of the fat-free mass and is responsible for 80% to 95% of glucose uptake at high insulin concentrations [30]. Therefore, glucose disposal following insulin injection could be better in the spirulina group with a higher fat-free mass. 

Soybean has controversial effects on glucose metabolism. Most animal studies have shown an improvement in insulin sensitivity and other parameters of glucose homeostasis [22,31,32,33,34]. However, one recently published study showed that in rats recovering from early life malnutrition that were fed on a soybean diet, there was an alteration in the insulin signaling pathway, leading to an increase in insulin resistance [35]. In humans, early studies done in diabetics registered an improvement in glycemic control [36], but recently published studies have not shown any beneficial effects on indices of glucose homeostasis [21,37]. These different results could be due to different study design, different daily intake and forms (fermented or not) of soybean protein and different study duration. In our study, insulin sensitivity improved within the soybean group (60% increase from baseline) but was significantly lower than the improvement observed in the spirulina group (167.7% difference between the two groups, *p* < 0.001). This indicates that, regardless of the true effect of soybean on glucose homeostasis in humans, spirulina seems to be superior to soybean in correcting HIV/HAART-associated insulin resistance.

No significant difference was noted in other anthropometric and the following biological parameters (CD4 count, TC and TG) measured between the two groups even though a trend of lower fat-free mass and TC were detected in the spirulina group after eight weeks (34.5 kg ± 11.1 *vs.* 36.9 kg ± 10.9, *p* = 0.59 and 2.38 g/L ± 0.67 *vs.* 2.65 g/L ± 0.67, *p* = 0.32, respectively).

In both groups, the fasting glycemia, TC and TG levels increased but this increase was greater in the soybean group than in the spirulina group. This suggests that spirulina seems to better retard the aggravation of HAART-induced hyperglycaemia and dyslipidaemia as opposed to soybean, as shown by the lower increases in FBG, TC and TG levels. 

**Table 3:** Change of other characteristics from baseline.

	Spirulina (*n* = 11)	Soya bean (*n* = 16)
Waist Circumference, mean (SD), cm	−0.40 (3.16)	+0.01 (2.34)
Total Body Fat, mean (SD), kg	+0.36 (4.52)	+0.38 (3.30)
Fat-Free Mass, mean (SD), kg	+0.35 (2.01)	−1.59 (1.57)
Fasting glycemia, mean (SD), g/L	+1.3 (0.9)	+1.8 (2.1)
Total cholesterol, mean (SD), g/L	+0.63 (0.53)	+0.87 (0.79)
Triglycerides, mean (SD), g/L	+0.48 (0.52)	+0.61 (0.70)

Abbreviation: *n*, number of subjects; SD, Standard deviation.

Although in this pilot study, spirulina intake was associated with a better improvement in insulin sensitivity than soybean intake, our results are limited by the small sample size and losses to follow up in the spirulina group (35%). Also, even though the groups were well balanced for factors that can influence insulin sensitivity at baseline like age, sex, type and duration of HAART exposure, BMI and WC, other factors like the presence or absence of hepatic steatosis and lipodystrophy were not investigated. Nevertheless, predictors for lipodystrophy (sex, type and duration of exposure to HAART) were comparable between the groups, indicating that lipodystrophy was also evenly distributed at baseline. Consequently, further studies that take these limitations into account are required to replicate our results.

## 4. Conclusions

In conclusion, spirulina supplementation in HIV patients may play a more beneficial role than soybean intake in improving on HIV/HAART associated insulin resistance. Further studies, involving more subjects and for a longer duration, are required to replicate these results and uncover the underlying mechanisms involved.
